# Endoscopic subcutaneous mastectomy: A novel and effective treatment for gynecomastia

**DOI:** 10.3892/etm.2013.1032

**Published:** 2013-04-02

**Authors:** HUA CAO, ZHI-XUE YANG, YI-HUI SUN, HAO-RONG WU, GUO-QIN JIANG

**Affiliations:** Department of General Surgery, The Second Affiliated Hospital of Soochow University, Suzhou, Jiangsu 215004, P.R. China

**Keywords:** gynecomastia, endoscopic, mastectomy

## Abstract

The aim of this study was to evaluate the procedure for and efficacy of endoscopic subcutaneous mastectomy for gynecomastia. Endoscopic subcutaneous mastectomy was performed on 100 benign, palpable breast enlargements in 58 male patients who were followed-up for 15–63 months. The surgery was conducted with the insufflation of CO_2_ subdermally. No cases were converted to open surgery. The unilateral surgery time was 70–90 min. The mean volume of the resected tissue was 200 ml. All procedures were completed successfully, with satisfactory clinical effects and ideal esthetic results postoperatively. There were three cases (3%) of papillary epidermal partial necrosis; following removal of the dressing during the hospital stay, normal nipple sensation returned. Endoscopic subcutaneous mastectomy had good clinical effects and ideal cosmetic results and is an appropriate approach for gynecomastia.

## Introduction

Gynecomastia is a benign abnormal enlargement of one or two breasts that is estimated to affect >40% of males (1). The treatment of gynecomastia depends on the underlying cause. In the majority of patients with pubertal gynecomastia, the condition resolves gradually (2,3). Medical treatment has been shown to be effective in the proliferative phase of gynecomastia. In a number of cases, however, fibrotic tissue develops and medical therapy is less helpful. When gynecomastia has been present for >2 years, medical therapy may no longer be effective and surgery may be the only useful treatment (3).

Traditional open surgery causes significant scarring, seriously affecting the appearance of the breast, so a number of patients refuse surgery for this reason. Although traditional surgery removes the hyperplasia completely, the results are cosmetically unsatisfactory in up to 50% of patients (4). Endoscopic subcutaneous mastectomy leaves fewer scars and causes no sensory disturbances. Using this technique, we treated 58 males with gynecomastia, with satisfactory therapeutic results.

## Patients and methods

### Patients

A series of 58 male patients who were diagnosed with Simon grade IIB benign gynecomastia or larger breasts, aged 17–52 years (average, 28 years), underwent endoscopic subcutaneous mastectomy in the Second Affiliated Hospital of Soochow University (Suzhou, China) between January 2006 and July 2010. Sixteen of the patients underwent a unilateral procedure and 42 had a bilateral excision; 42 breasts presented as cylindrical cones and 10 exhibited mild ptosis. The breast diameter ranged from 10 to 16 cm and the height from 4 to 6 cm. Fifty-six breasts presented little development of the nipple. The history of gynecomastia ranged from 13 months to 12 years. In all patients, medical therapy had failed and surgical treatment was required since the problem was causing discomfort and affecting their daily lives. All patients underwent pre-operative mammography to assist in diagnosis and breast enlargement caused by simple obesity was excluded. This study was conducted in accordance with the Declaration of Helsinki and with approval from the Ethics Committee of the Second Affiliated Hospital of Soochow University. Written informed consent was obtained from all participants.

### Surgical procedure

**Figure 1:**
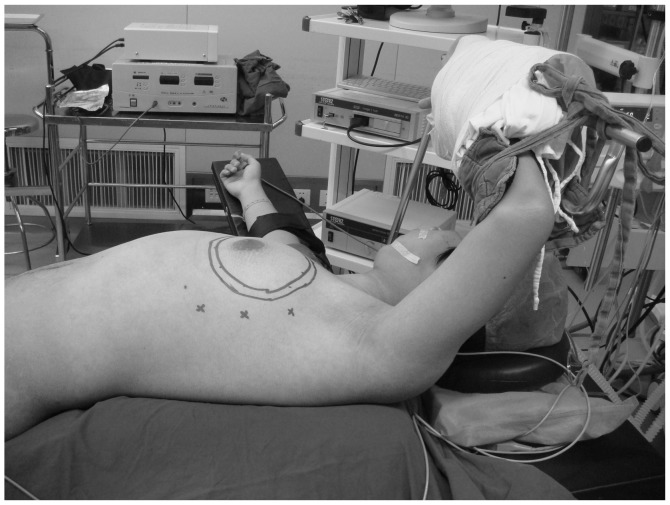
Position of patient during surgery: supine position with the ipsilateral limb wrapped around the head rack, with a thin pillow inserted under the back on the operative side.

All patients underwent general anesthesia and were placed in the supine position with the ipsilateral limb wrapped around the head rack and a thin pillow inserted under the back on the operative side (Fig. 1). This position was optimal for the use of the ultrasonic scalpel and assistant clamp. The breast enlargement was marked on the patients’ skin pre-operatively; a 1-cm mark outside the enlargement was also marked to determine the area of resection.

**Figure 2:**
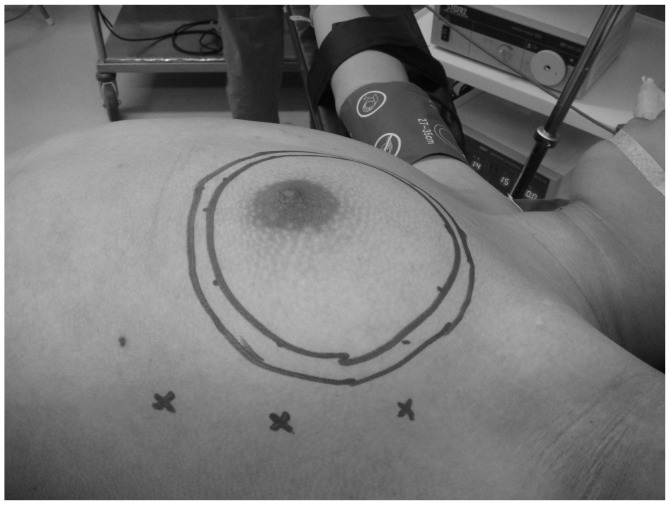
Markings showing breast enlargement and an area 1 cm beyond this (resection area); position of placement of the three trocars.

**Figure 3:**
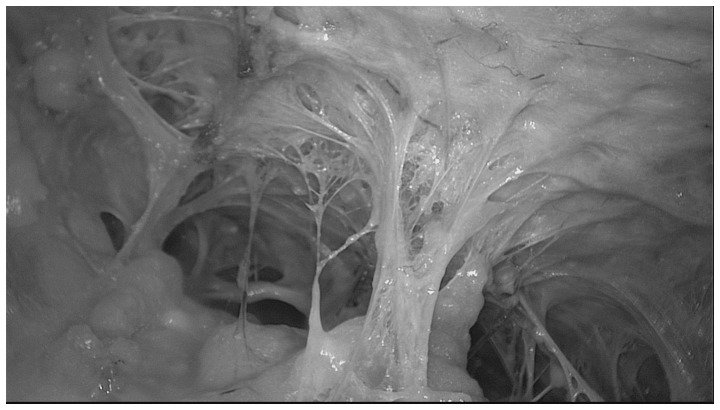
Dissolving fat on endoscopy.

Three small skin incisions were made on the mid-axillary line (Fig. 2). The middle incision was 10 mm in length and the other two were 5 mm. The 10-mm incision was made at the intersection of the mid-axillary line and the horizontal line through the nipple; the 5-mm incisions were 5 cm superior and inferior to this (Fig. 2). Warm fat dissolving solution (200 ml 0.9% saline, 200 ml distilled water, 0.5 mg epinephrine and 20 ml 2% lidocaine) was injected into the incisions and allowed to spread evenly throughout the entire breast subcutaneous tissue and posterior breast space. After 20 min, all dissolved subcutaneous and posterior breast space adipose tissue, with the exception of the fat tissue under the areola, was extracted by vacuum suction (Fig. 3). The subcutaneous fibrous septum was detached as much as possible under direct vision; then, using the little finger as a guide, a trocar with an inner diameter of 10 mm was placed in the middle incision. Through this trocar, a space was established by the insufflation of CO_2_ at a pressure of 6–8 mmHg. Two 5 mm inner diameter trocars were placed in the upper and lower incisions, respectively, and the endoscope was placed in the middle trocar.

**Figure 4:**
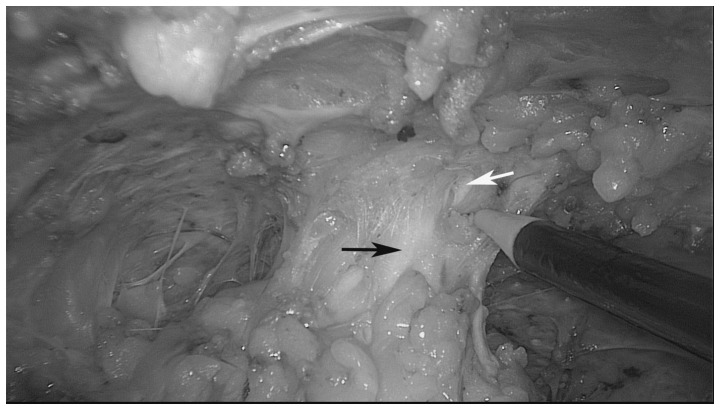
Breast tissue pressed downward (black arrow) and nipple-areola complex drawn upward (white arrow).

A cutting hemostasis ultrasonic scalpel and assistant clamp were inserted into the two incisions to subdermally dissect the fibrous septum. When operating near to the areola, the nipple-areola complex was drawn upward and the breast tissue was pressed downward to avoid injury (Fig. 4). The thickness of the complex was maintained at ∼1 cm; otherwise, partial necrosis may occur postoperatively. The extent of dissection of the skin overlying the breast was that marked preoperatively on the breast mound.

**Figure 5:**
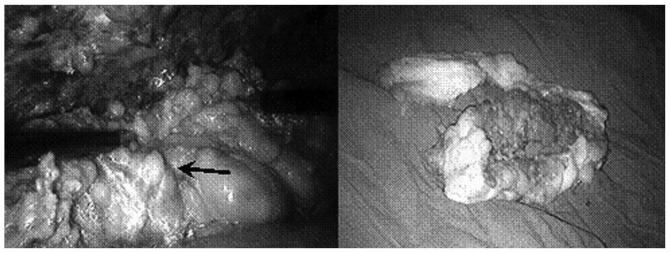
The whole breast underwent en bloc resection. The arrow indicates breast tissue. The volume of the resected tissue was 300 ml.

**Figure 6:**
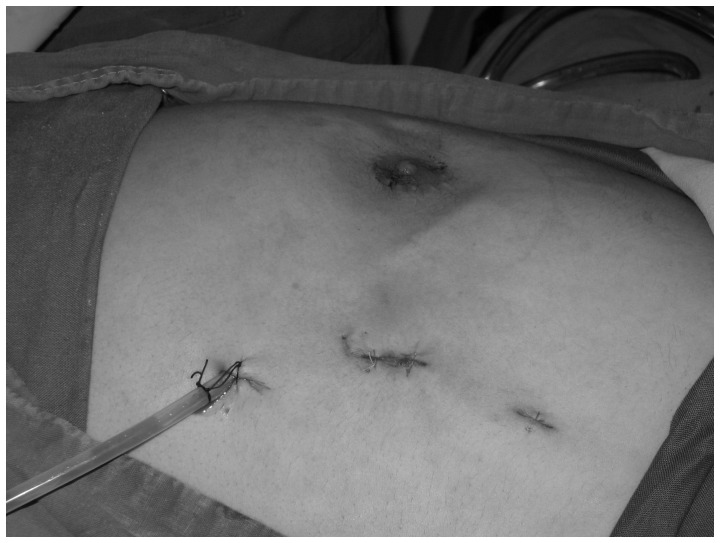
Postoperative appearance: sutures and drainage.

**Figure 7:**
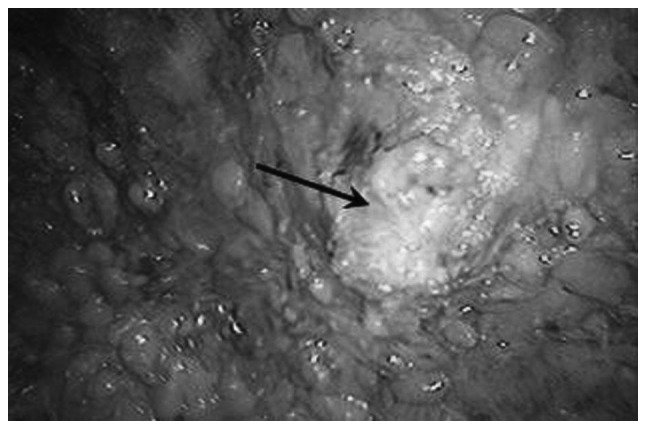
Nipple pressed downwards following complete resection. The arrow indicates the centre of the nipple.

After the breast tissue had been fully separated from the overlying skin, a prepectoral fascia separation was performed from the axillary side. The whole breast underwent en bloc resection with the ultrasonic scalpel and was removed (Fig. 5). Physiologic saline was used to irrigate the wound and achieve thorough hemostasis (Fig. 6). A suction drain was brought out through the lowest incision. All the incisions were closed with interrupted absorbable sutures (Fig. 7). An elastic compression bandage was used to wrap the wound. The resected tissue was placed in a 500-ml measuring cup that was then filled with water. After removing the tissue, the remaining water volume was measured to calculate the tissue volume (tissue volume = 500 ml − remaining water volume).

## Results

**Figure 8:**
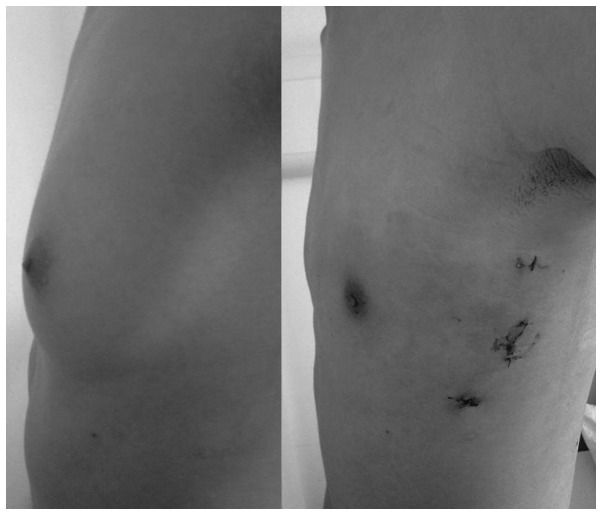
Comparison of appearance pre- and post-operatively.

All procedures were completed successfully. The initial unilateral surgery time was 100–150 min, which has been improved to 70–90 min with experienced surgeons and improved cooperation. The volume of the resected specimens ranged from 150 to 300 ml, with a mean of 200 ml. There were no cases of accidental damage or conversion to open surgery and no significant bleeding. Postoperative drainage from one side was ∼20 ml per day and the drainage tube was removed 2–3 days after surgery. No subcutaneous fluid collections occurred. There were three cases of papillary epidermal partial necrosis; however, after the dressing was removed during the hospital stay, normal nipple sensation resumed. There was no epidermal necrosis at the incision sites and all patients achieved satisfactory clinical effects and ideal cosmetic results. The duration of follow-up was 15–63 months, during which no numbness or other postoperative complications occurred and a satisfactory breast form was achieved (Fig. 8).

## Discussion

Gynecomastia is caused by physiological and pathological factors. In young patients, particularly adolescents, the causes are mainly physiological at puberty and the gynecomastia usually resolves spontaneously. In the current study, the majority of patients that we selected had passed puberty, which meant that the condition was unlikely to resolve spontaneously. Secondly, prior to surgery, all patients underwent breast mammography to ensure that the mammary gland itself was significantly enlarged. In the present study, the mean volume of resected breast tissue was 200 ml, with a maximum of 300 ml. The indication for endoscopic resection was breast enlargement at Simon grade IIB or above, with glandular hyperplasia. Thirdly, the longest duration of disease in this group was 12 years and the shortest treatment time of patients within this group was 13 months. Therefore, all patients in this study had been through a long-term medical consultation process and drug therapy before they underwent endoscopic subcutaneous mastectomy.

Gynecomastia is considered to be present in >30% of males, with much higher rates in males aged >70 years. We elected not to select elderly patients in this study for several reasons. Firstly, gynecomastia in elderly male patients is often caused by declining levels of testosterone. Treatments with additional androgens and traditional Chinese medicine may have therapeutic effects. Secondly, elderly patients tend not to be as concerned with scars on the chest and are more likely to choose a traditional surgical resection approach. Finally, elderly patients are less likely to take on the relatively high costs of this new technique compared with the standard surgical approach used in China.

**Figure 9:**
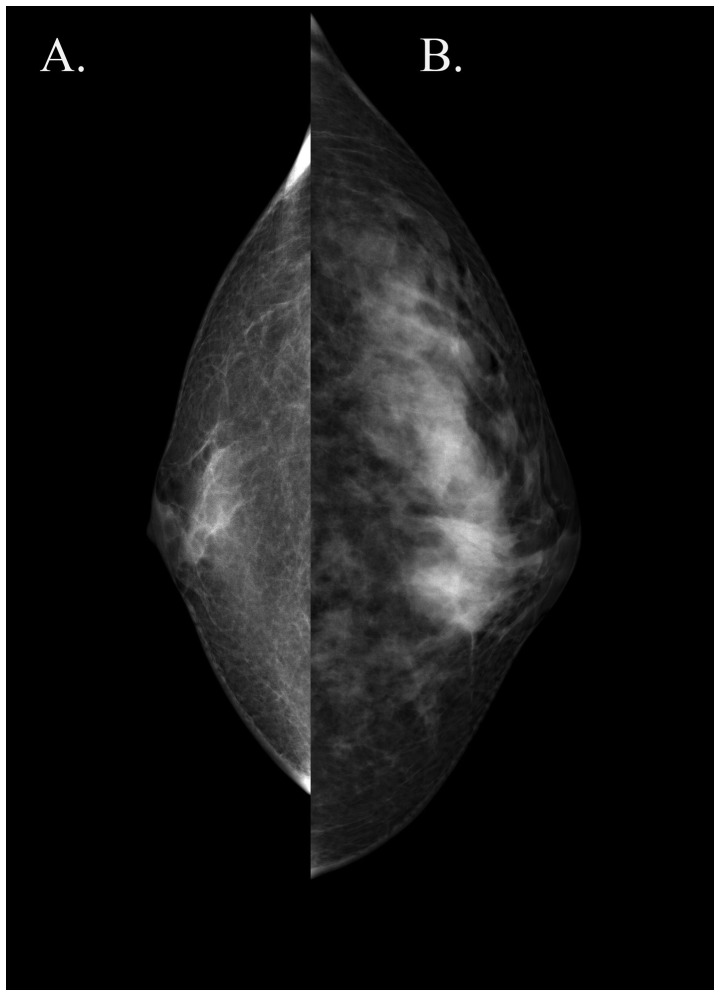
(A) Increase in breast mass due to the accumulation of adipose tissue, which may be treated by liposuction and periareolar incision. (B) Enlargement of the breast is caused by mammary gland enlargement and only endoscopic surgery is suitable.

Obesity in males often causes increased breast size and yet this increase is simply due to the accumulation of adipose tissue. Thus, before the decision is made to perform surgery, we ensured that every patient underwent breast mammography to ensure that the breast enlargement was caused by an increased mammary gland size rather than increased volumes of adipose tissue (Fig. 9). If the mammary gland and adipose volume had increased, in general we advised patients to lose weight first and then have surgery, as the surgical outcomes are better in patients with a lower level of adiposity.

The surgical methods for the treatment of gynecomastia include liposuction, periareolar incision and endoscopic surgery. Mastectomy with a periareolar incision removes a Simon grade I or II enlarged breast; however, longer incisions are required for breasts that are grade IIB or larger. These incisions leave large scars on the chest, which are often unsatisfactory to the patient (5). Liposuction removes smaller amounts of fatty tissue; however, it does not work well when there is a firm glandular component (6). Liposuction plus local resection through a periareolar or remote incision has replaced open excision in cases of gynecomastia with severe hypertrophy (7,8), significant skin excess and grade 3 ptosis. In the current study, we selected patients with larger developed breasts, with a mean volume of 200 ml of resected breast tissue. To resect large breasts, periareolar incision surgery is likely to leave visible scars and liposuction is unable to achieve a complete resection (Fig. 9). Endoscopic microsurgery with a 10 mm incision at the mid-axillary line has satisfactory esthetic results that are welcomed by the majority of patients. In the present study, we proposed points to achieve a good surgical effect, as follows: i) the dissolving and suction of all fat tissue, with the exception of that under the nipple and areolar area, provides easy access for the separation of breast and subcutaneous tissue; ii) the subcutaneous fibrous septum should be detached as much as possible under direct vision; using the little finger as a guide, the trocar may then be placed properly; iii) during subdermal breast parenchyma dissection, when operating near the areola, the nipple-areola complex should be drawn upward and cuts made slowly to avoid injury. Damage to the vasculature of the complex may be avoided if some glandular tissue remains. It is important to keep the thickness of the complex at ∼1 cm, to reduce the risk of postoperative nipple necrosis; and iv) the sequence of the procedure is important. We detached the subcutaneous fibrous septum to provide a satisfactory working space for the glandular resection. The resected tissue was then pulled out of the trocar hole with no need to lengthen the incision, improving the esthetic results.

It should be noted that, during insufflation, too high a pressure causes pneumoderma and too low a pressure may induce respiratory movements that disturb the surgery. Thus, we suggest that the pressure of insufflation of CO_2_ should be 6–8 mmHg. Three cases of partial necrosis of the nipple area were identified. These may have resulted from injury to the subdermal vasculature or heat damage to the nipple skin from the ultrasonic scalpel.

Liposuction plus ultrasound-guided vacuum-assisted breast biopsy is a recently reported, potential method for esthetic surgery (9,10). However, the endoscopic resection of an enlarged breast achieves the complete and accurate removal of mammary tissue under direct vision with clear advantages.

In conclusion, endoscopic subcutaneous mastectomy for gynecomastia had satisfactory esthetic results and no significant postoperative complications. It is an appropriate surgical approach for gynecomastia, providing that skilled operators and suitable endoscopic technology are available.
